# Supplementary material for: Exercise induces new cardiomyocyte generation in the adult mammalian heart
Source: Nat Commun. 2018 Apr 25;9:1659. doi: 10.1038/s41467-018-04083-1 (PMC5916892; doi:10.1038/s41467-018-04083-1)
Supplement: Supplementary file 1 — Supplementary Information [file 41467_2018_4083_MOESM1_ESM.pdf]

Supplementary Materials for

Exercise induces new cardiomyocyte generation in the adult mammalian heart

Vujic and Lerchenmüller et al.

Supplementary Figure 1

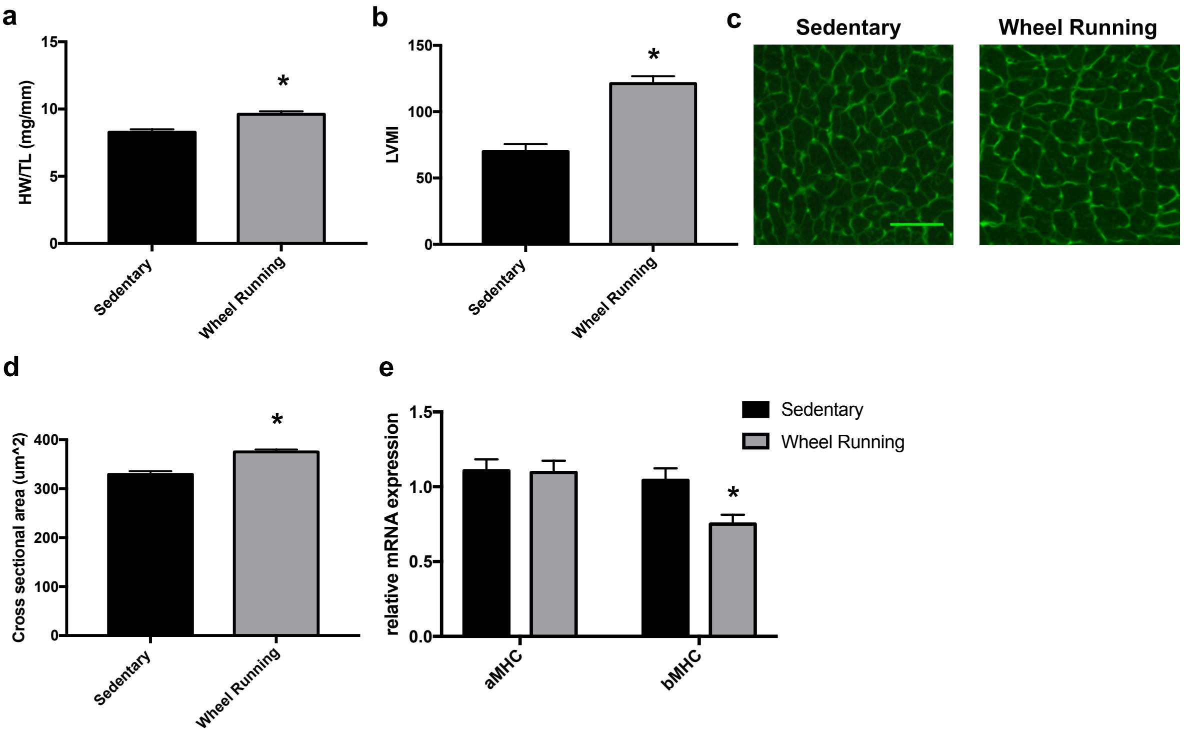

Supplementary Figure 1. Voluntary wheel running exercise induces cardiac hypertrophy in young mice.

a Heart weight to tibia length ratio was significantly increased in young adult mice when compared to aged-matched sedentary animals (n=8 mice/group, *p<0.05, student's t-test). **b** Echocardiographic analysis of the left ventricular mass index (LVMI) from exercised and age-matched sedentary animals (n=4 mice/group, *p<0.05, student's t-test). **c-d** Representative images of cardiomyocyte Cross Sectional Area (CSA) analyzed from wheat-germ-agglutinin stained transverse heart sections. Scale bar = 50μm 300 cardiomyocytes were counted per group (n=3 mice/group, *p<0.05, student's t-test). CSA increases 14% in response to exercise. **e** Anticipated switch of alpha/beta myosin heavy chain gene expression after voluntary wheel running (n=4 mice/group, *p=0.05, student's t-test). Results are presented as mean ± s.e.m.

Supplementary Figure 2

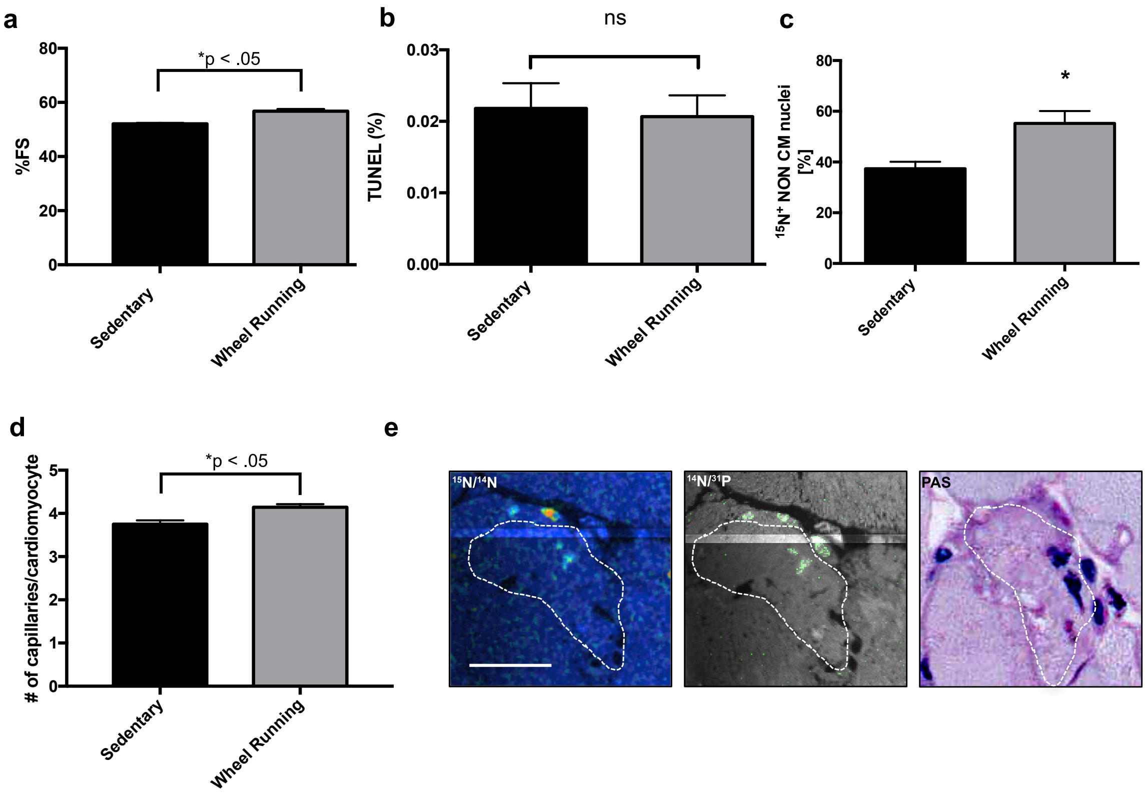

Supplementary Figure 2. Cardiomyogenesis and cardiac growth in response to exercise is due to physiologic and not pathologic remodeling. a Echocardiographic analysis shows an improved fractional shortening (%FS) in exercised and age-matched sedentary animals (n=4 mice/group, *p<0.05, student's t-test). **b** Quantification of apoptotic cardiomyocytes by TUNEL staining (>14,000 nuclei were counted from 3-5 mice/group, p=ns, student's t-test). **c** Exercise increases $^{15}N^{+}$labeled non-cardiomyocyte fraction (sedentary: exercised = 37:55%) (>4000 non-cardiomyocytes from four mice/group were counted, *p<0.05, student's t-test). **d** Number of capillaries measured by CD31 positive capillaries per cardiomyocyte in transverse heart sections increased by 10% after voluntary wheel running in comparison to matched control sedentary mice (n=3 mice/group, *p<0.05, student's t-test). **e** Representative images of a ^{15}N-labeled cardiomyocyte nuclei undergoing cytokinesis. Mass image $^{14}N/^{31}P$ (left), HSI mosaic tile $^{15}N/^{14}N$ (center) and adjacent section with Periodic acid Schiff staining (PAS)(right). The color scale in the HIS image ranges from blue, where the ratio is equivalent to natural ratio (0.37 %, expressed as 0% above natural ratio (enrichment over natural ratio)), to red, where the ratio is 150 % above natural ratio. Scale bar = 25μm). Results are presented as mean ± s.e.m.

Supplementary Figure 3

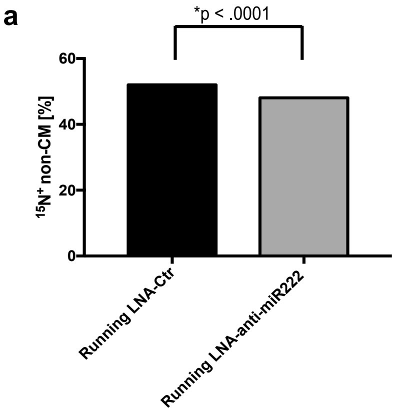

Supplementary Figure 3. Inhibition of miR222 reduces non-cardiomyocyte DNA synthesis

a Mice (2 months old) treated with LNA-anti-miR222 undergoing voluntary wheel running show a reduction in non-cardiomyocyte DNA synthesis (n=4 mice/group, *p<0.0001 Running LNA-scr-miR vs Running LNA-anti-miR222, Fisher's exact test).
